# Influence of initial conditions on absolute and relative dispersion in semi-enclosed basins

**DOI:** 10.1371/journal.pone.0217073

**Published:** 2019-07-26

**Authors:** Francesco Enrile, Giovanni Besio, Alessandro Stocchino, Marcello G. Magaldi

**Affiliations:** 1 Dipartimento di Ingegneria Civile Chimica e Ambientale, Università degli Studi di Genova, Genova, Italia; 2 ISMAR-CNR, S.S. di Lerici, Lerici, SP, Italia; 3 Johns Hopkins University, Earth and Planetary Sciences, Baltimore, MD, United States of America; University of Guam, GUAM

## Abstract

Absolute and relative dispersion are fundamental quantities employed in order to assess the mixing strength of a basin. There exists a time scale called Lagrangian Integral Scale associated to absolute dispersion that highlights the occurrence of the transition from a quadratic dependence on time to a linear dependence on time. Such a time scale is commonly adopted as an indicator of the duration needed to lose the influence of the initial conditions. This work aims to show that in a semi-enclosed basin the choice of the formulation in order to calculate the absolute dispersion can lead to different results. Moreover, the influence of initial conditions can persist beyond the Lagrangian Integral Scale. Such an influence can be appreciated by evaluating absolute and relative dispersion recursively by changing the initial conditions. Furthermore, finite-size Lyapunov exponents characterize the different regimes of the basin.

## 1 Introduction

**Fig 1:**
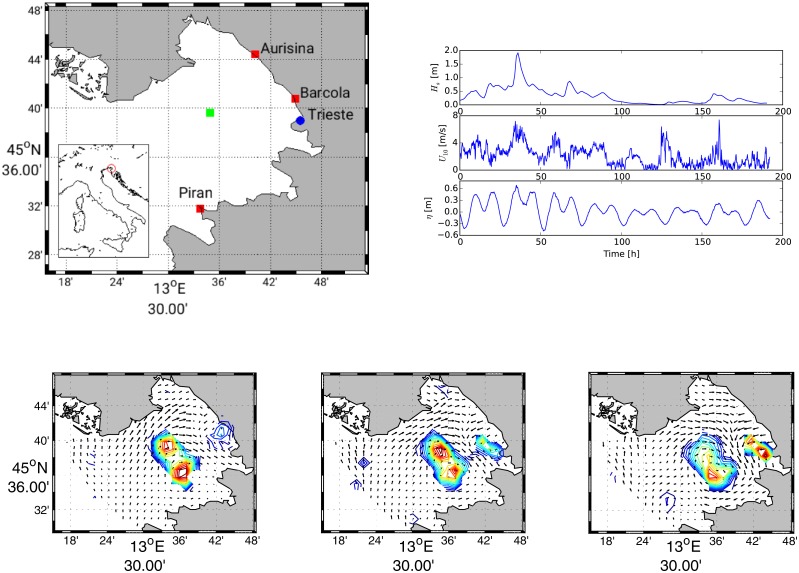
Top panels: Geographical map showing the location of the Gulf of Trieste and the position of the HF-radar (red squares), the tidal gauge and anemometer station in Trieste (blue circle) and the point of sea wave reanalysis (green square). Line plots reporting the significant wave height *H*_*s*_ from reanalysis [10], wind velocity *U*_10_ from the Trieste anemometer station and water level from tidal gauge in the port of Trieste (source data from www.mareografico.it) for the same time frame of the HF-radar dataset. Bottom panels: 2D plots of the HF-radar surface total velocity fields with superimposed contours of swirling strength that show vortex merging.

The Gulf of Trieste is a shallow semi-enclosed basin in the NE Adriatic Sea (see Fig 1) with a maximum depth of 25 m. The circulation is usually cyclonic and the main forcings are the Istrian coastal current at the southern border and the tides. However, an east to west current at the surface layer is produced by intense and frequent wind conditions coming from the north-eastern quadrant [1]. Pronounced seasonal cycles result in variable oceanographic properties with thermal stratification during summer and the formation of strong salinity gradients originated by the contrasting effects of fresh water runoffs and seawater exchange at the open boundary [2]. Tidal oscillations enter in the Adriatic Sea from the Otranto strait [3]: the dominant tides are diurnal and semidiurnal. Tidal currents can enhance horizontal dispersion [4] and their footprints should be clearly identifiable in Lagrangian statistics. The aim of this work is to detect such footprints and to verify which formulation is best suited in order to evaluate absolute dispersion. Absolute dispersion measures the spread of tracers released in a water body. We will see in the following paragraphs that such a spreading can be evaluated by following two different approaches. The behaviour of absolute dispersion is intrinsically related to the Lagrangian Integral Time *T*_*L*_, i.e. the integral of the auto-correlation function of residual velocities. This time defines the occurrence of the change in the slope of the absolute dispersion plot. For times smaller than *T*_*L*_, the absolute dispersion has a quadratic dependence on time whereas, for times larger than *T*_*L*_, a linear dependence on time is expected. We aim to evaluate such statistics varying the initial conditions, i.e. the time instance at which numerical particles are released and, consequently, the velocity at which they are subjected at the beginning of their trajectory. Indeed, the evaluation of the absolute dispersion varying the initial conditions can lead to results having one order of magnitude of difference, as it will be shown in the next paragraphs. Even *T*_*L*_ could be influenced by initial conditions. However, the present case does not show any important impact over the computation of *T*_*L*_. Therefore, there is the need to further evaluate the time scale over which the influence of the initial conditions is lost.

## 2 Observations: Methods and datasets

In the last years a network of HF-radars has been installed in the gulf of Trieste as part of a European cooperation project: Tracking Oil Spill and Coastal Awareness Network. The HF-radar network consists of three monostatic CODAR SeaSonde systems (Fig 1) and provides a complete coverage of the gulf. The working frequency was set to 25 MHz, bandwidth to 150 kHz, for a radial resolution of 1 km with a spatial extension of about 30 km, with an angular resolution of 5° and employs the MUSIC (MUltiple SIgnal Classification) direction finding algorithm [5] to derive radial currents on a hourly basis. It is worth mentioning that during the same project a drifter experiment was carried out, releasing 41 CODE (Coastal Ocean Dynamics Experiment) drifters [6, 7]. The Lagrangian velocities measured from the drifters’ trajectories have been used to validate the radial and total velocities observed with the HF-radar network, see for details [8] and [9].

For the present analysis, a time frame from 23rd April and 30th April 2012 will be used for the computations. Wind velocities *U*_10_ and water level *η* recorded from the tidal gauge of Trieste (source data from www.mareografico.it) are compared with the significant wave height *H*_*s*_ calculated from reanalysis [10] for the time frame of interest in Fig 1. Water level oscillations are of the order of 0.6m and they can be enhanced by the effects of wind set-up.

## 3 Data analysis: Lagrangian integral time scales and dispersion computation

The starting point of our Lagrangian analysis are numerical particle trajectories, described by the time-dependent position ***x***(*t*), computed from the HF-radars total surface velocity fields in an Eulerian framework. Particle trajectories are calculated using a fourth-order Runge–Kutta algorithm with adaptive step size employing a bi-cubic spatial interpolation and a polynomial time interpolation of the experimental Eulerian field. This is an approach commonly adopted in mixing studies [11]. Then, the numerical trajectories will be employed in the evaluation of single and multiple-particle Lagrangian statistics, leading to the estimation of absolute and relative dispersion or Finite Size Lyapunov Exponent. Particles are released on a regular grid that covers all the Gulf, disregarding the boundaries. The spacing is equal to 100 m in both directions.

Regarding the absolute dispersion ***A***^2^(*t*), let us assume to deal with a certain number of particle trajectories released at some initial time *t*_0_. The subsequent computation of single-particle statistics could be performed considering or not the displacement of the centre of mass of the cluster of particles (namely a mean drift). In the latter case, what matters are only the initial conditions and its computation can be performed following the definition of [12], already applied in several geophysical contexts, see [13] and [14] among others. Then, it is common to consider the mean-square displacement given by the trace of ***A***^2^, usually referred to as the total absolute dispersion *a*^2^(*t*) and its time derivative as the total absolute diffusivity *K*^(1)^(*t*):

$$\begin{array}{c} A_{ij}^{2}\left(t\right)=\frac{1}{M}\sum_{m=1}^{M}\{[x_{i}^{m}\left(t\right)-x_{i}^{m}\left(t_{0}\right)][x_{j}^{m}\left(t\right)-x_{j}^{m}\left(t_{0}\right)]\}\;a^{2}\left(t\right)=A_{xx}^{2}\left(t\right)+A_{yy}^{2}\left(t\right) \end{array}$$ (1)

where M is the number of particles and ***x***^*m*^(*t*) is the position of the *m*-th particle at time *t* and ***x***^*m*^(*t*_0_) is its initial position.

Typical dispersion regimes are identified from the time-dependence of *a*^2^(*t*). Two regimes are usually detected: for times smaller than the Lagrangian time scale *T*_*L*_ an absolute dispersion function of square time is recovered, whereas for times greater than *T*_*L*_ a linear dependence from time is obtained:

$$\begin{array}{c} A_{ii}^{2}\left(t\right)=\rho_{L_{ii}}\left(0\right)t^{2}\;t<T_{L_{i}}\;\;A_{ii}^{2}\left(t\right)=2\rho_{L_{ii}}\left(0\right)T_{L_{i}}t+\text{const.}\;t>T_{L_{i}} \end{array}$$ (2)

where $T_{L_{i}}$ is calculated as the time integral of the Lagrangian autocorrelation function of the *i*-th velocity component as [15]

$$\begin{array}{c} T_{L_{i}}=\int_{0}^{+\infty }R_{ii}dt\;R_{ii}\left(\tau \right)=\frac{1}{M}\sum_{M}\frac{\rho_{L_{ii}}\left(\tau \right)}{\sqrt{\rho_{L_{ii}}\left(0\right)\rho_{L_{ii}}\left(0\right)}}\;\rho_{L_{ii}}\left(\tau \right)=\left\langle u_{L_{i}}^{\prime }\left(t\right)u_{L_{i}}^{\prime }\left(t+\tau \right)\right\rangle . \end{array}$$ (3)

where the brackets indicates an average over the entire duration of each trajectory and $u_{L_{i}}^{\prime }$ is the *i*-th Lagrangian component of the residual velocity. Note that the absolute diffusivity is the product of the Lagrangian time scale and the velocity variance. From the above formulation it clearly appears that a correct definition of the residual velocity is of crucial relevance. This issue has been extensively discussed in the oceanographic literature and, in particular, whenever Lagrangian statistics have been based on Lagrangian observations [16–21]. A common way to evaluate residual velocities consists in subtracting from the Lagrangian velocity of a particle an Eulerian mean velocity ***U***(*x*, *y*), defining the residual velocities as $u_{L}^{\prime }\left(x,y,t\right)=u_{L}\left(x,y,t\right)-U\left(x,y\right)$. We return on this issue in the next section.

A different approach in the evaluation of the absolute dispersion involves the computation of a mean drift related to the center of mass of a cluster of particles ***M***(*t*) [13]. The total absolute dispersion (in this case indicated with *d*^2^(*t*)) is therefore evaluated removing at each time the position of the centre of mass. In the following section, we will apply both methods to estimate absolute dispersion, discussing the differences in the final results.

Moreover, we are interested in investigating how a pair of particles tend to spread in time and space in response to the flow structures at several scales. This analysis will be performed calculating different Lagrangian measure based on multiple particles statistics, such as relative dispersion and Finite Size Lyapunov Exponent (FSLE). Regarding the former, relative dispersion ***R***^2^(*t*) is defined as the mean-square distance at time *t* between a pair of particles that at time *t*_0_ have a distance equal to *r*_0_ [22–24]:

$$\begin{array}{c} R_{ij}^{2}\left(t\right)=\frac{1}{M-1}\sum_{m=1}^{M-1}\{[x_{i}^{m}\left(t\right)-x_{i}^{m+1}\left(t\right)][x_{j}^{m}\left(t\right)-x_{j}^{m+1}\left(t\right)]\} \end{array}$$ (4)

As before the total relative dispersion *r*^2^(*t*) is simply the trace of the relative dispersion matrix and the total relative diffusivity *K*^(2)^(*t*) is its time derivative. Instead, FSLE Λ(*r*) are calculated averaging times rather than separations. Thus, FSLE curves are calculated setting separations that increase recursively as *r*_*n*_ = *αr*_*n*−1_, where *α* is equal to 1.2, and averaging the times *τ*_*n*_ needed for particle pairs to reach such separations. Therefore, FSLE Λ(*r*) are defined as:

$$\begin{array}{c} \Lambda \left(r\right)=\frac{1}{\left\langle \tau_{n}\right\rangle }\;\text{log}\;\alpha \end{array}$$ (5)

where brackets represents the average.

## 4 Results

### 4.1 Lagrangian integral time scale: The role of the mean flow and tidal oscillations

The analysis of surface dispersion is inherently related to the spatial and time flow scales. In particular, the absolute dispersion is strongly related to the Lagrangian time scales as shown in Eq 2. Therefore, we firstly discuss the results regarding the velocity auto-correlation and Lagrangian time scales. As mentioned in the methodological section it is crucial to define the proper field of residual velocities or, in other words, a proper “mean velocity”. A possible approach employed starting from Lagrangian observations derived from oceanic drifter experiments, was to calculate the mean flow by averaging the velocities of the drifters having subdivided the domain in geographical bins. The binning method has been widely applied, e.g. in the North Atlantic by [19], in the Nordic Sea by [25] and Tropical Pacific by [20], among others. However, several drawbacks have been identified in the binning method and improvements have been suggested during the last years [16–18, 21]. In particular, critical points are the actual number of drifters that pass through each bin, the size of the bin itself that should be related to the scale of the mean flow to be captured and the uneven distribution of the trajectories. Note that the mean flow affected by potential errors may lead to an erroneous evaluation of important quantities such as the Lagrangian integral scales. In the present study, we suggest a different approach to compute the residual velocity. Indeed, the Eulerian observations derived for the HF-radar perfectly fit for this goal, owing to a relatively high temporal acquisition frequency and an acceptable spatial resolution. Therefore, the Eulerian mean velocity ***U***(*x*, *y*) is easily recovered by averaging the time-signal of the recorded velocity for each grid node of the domain covered by the HF-radar network. Then, a spatial nearest-neighbour interpolation is employed in order to associate to the velocity of the particle at each time-instance the corresponding Eulerian mean velocity of the nearest node. The residual velocity signals are used to compute autocorrelations and Lagrangian integral time scales. We evaluated ***U***(*x*, *y*) over the duration of the dataset at our disposal. However, an analysis based on a longer dataset that could evaluate such quantity on a seasonal base would be beneficial for a climatological analysis.

**Fig 2:**
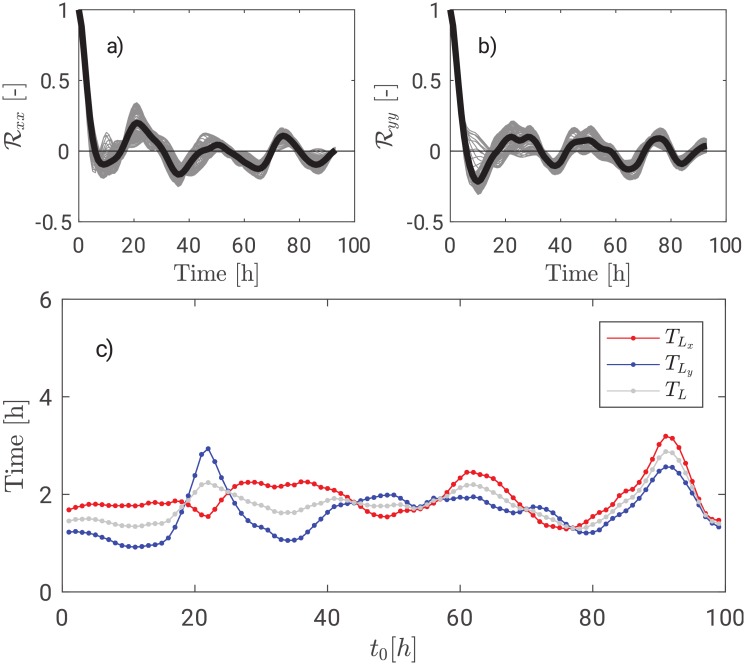
Top panels: Lagrangian autocorrelation functions $R_{xx}(\tau )$ and $R_{yy}(\tau )$. Bottom panel: Lagrangian time scales $T_{L_{x}}$, $T_{L_{y}}$ and their average *T*_*L*_.

The surface velocity fields, derived from the HF-radar observations, are known in definite time frame. Therefore, the subsequent Lagrangian analysis, based on numerical trajectories, requires the definition of an “initial time”. Typically, the initial time is set to coincide with the starting time of the available dataset. In case of quasi-steady forcing of the surface circulation, the discussion whether this choice is correct would be meaningless, owing to the stationary statistics of the flow. However, in the framework of the present work, we carried out recursively the computation of trajectories, autocorrelation and, finally, single and multiple particle statistics, changing the initial time of particle releases. This procedure is adopted in order to assess whether the field observed is statistically stationary or not. Fig 2a and 2b shows Lagrangian autocorrelation function for both *x* and *y* components. Grey curves are evaluated at the varying of the initial conditions whereas the black curve represent the average *μ*(*t*) = 〈*a*^2^(*t*)〉, where the average is carried out over the curves by fixing the time instance. Auto-correlations do not tend monotonically to zero and show a persistent, even if small, correlation even for long times. Should we have at our disposal a longer dataset, these fluctuations would probably disappear. Fig 2c shows the *x* and *y* Lagrangian time scales plotted against the initial condition. The Lagrangian time scale can be generalized as $T_{L}=\frac{1}{2}\left(T_{L_{x}}+T_{L_{y}}\right)$. The average over all the initial conditions is almost 2h. $T_{L_{x}}$ is persistently higher than $T_{L_{y}}$ due to the open boundary of the Gulf of Trieste on the Adriatic Sea whose orientation form an angle greater than 60° with the *x*-axis.

The Lagrangian time scale *T*_*L*_ is therefore quite independent from the initial conditions *t*_0_, i.e. its order of magnitude does not vary. The following paragraph will assess whether such an independence is also present in the absolute dispersion.

### 4.2 Absolute dispersion: The influence of the initial conditions and the introduction of a decorrelation time scale

**Fig 3:**
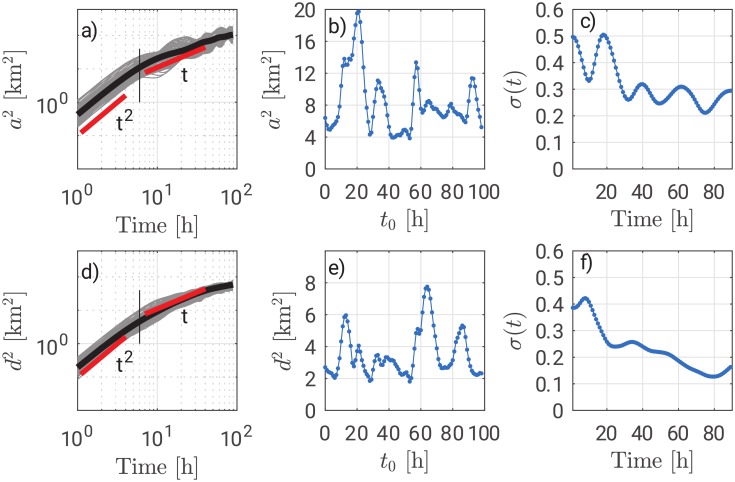
Panel a) shows total absolute dispersion *a*^2^(*t*). Panel b) shows total absolute dispersion along the section represented as a thin black line in Panel a). Panel c) shows the normalized standard deviation of the total absolute dispersion. Panels d), e) and f) show analogue quantities referred to the absolute dispersion about the center of mass. Note that Panels a) and d) are plotted on a log-log scale and therefore the absolute difference of two values belonging to different grey curves evaluated at the same time instance have one order of magnitude of difference.

While the Lagrangian time scale is quite robust at the varying of the initial conditions, the absolute total dispersion shows at least one order of magnitude of difference between the curves, suggesting that the results are not independent from the initial time. Such a dependence is the result of the tidal forcing whose influence on the absolute dispersion is the generation of a periodic signal. Fig 3a shows the total absolute dispersion *a*^2^(*t*) as a function of time. Grey curves are evaluated at the varying of the initial conditions and the dark curve represents the average. Red curves represent theoretical behaviours. Such behaviours are almost followed by the average. The thin black vertical line shows the section along which the values of total absolute dispersion are plotted on Panel b). The x-axis of Panel b) represents the initial condition at which the value of *a*^2^ refers to and must not be confused with the x-axis of Panel a). Panel b) shows an oscillating curve with a period of the order of 24h that can be associated with the diurnal tide. Therefore, the footprints of tidal oscillations are recovered as expected.

Furthermore, Panel a) shows that the grey curves tend to collapse one over each other for long times. For this reason, it is possible to quantify whether the curves tend to narrow by adopting a normalization with respect to the average *μ*(*t*). Then, it is possible to evaluate the standard deviation of the normalized values for each time *σ*(*t*) = std[*a*^2^(*t*)]/*μ*(*t*) over the computations of *a*^2^(*t*) with different initial conditions. Fig 3c shows that such a quantity tends to decrease as time elapses. Such a result is physically consistent since the particles tend to spread reaching separations that have on order of magnitude comparable to the length of the basin. Indeed, velocity autocorrelations are small when such separations are reached and FSLE Λ(*r*) curves present a subdiffusive regime (see next paragraph). Therefore, grey curves actually tend to collapse relatively to the average. Such a result suggests that the relative importance of the initial condition decreases as time elapses. As the Lagrangian time scale *T*_*L*_ shows when there is a change of regime on the plot of the total absolute dispersion, the function *σ*(*t*) evaluates the influence of the initial conditions on the strength of the absolute total dispersion after a time *t*. In case the function *σ*(*t*) would not decrease, the influence of the initial conditions would persist even for long times.

While autocorrelations and Lagrangian integral time scales are calculated once for all, the evaluation of absolute dispersion can be pursued taking into consideration the mean drift [13, 26]. The mean drift can be calculated as

$$\begin{array}{c} M_{i}\left(t\right)=\frac{1}{M}\sum_{m=1}^{M}[x_{i}\left(t\right)-x_{i}\left(t_{0}\right)] \end{array}$$ (6)

The spread about the center of mass can be measured as

$$\begin{array}{c} D_{i}^{2}\left(t\right)=\frac{1}{M-1}\sum_{m=1}^{M}[x_{i}\left(t\right)-x_{i}\left(t_{0}\right)-M_{i}\left(t\right){]}^{2} \end{array}$$ (7)

The total absolute dispersion about the center of mass can be evaluated as

$$\begin{array}{c} d^{2}\left(t\right)=D_{x}^{2}\left(t\right)+D_{y}^{2}\left(t\right) \end{array}$$ (8)

Fig 3d shows *d*^2^(*t*) as a function of time. Compared with of Fig 3a, the solution is smoother and grey curves tend to oscillate less. By adopting the definition related to the centre of mass, it is possible to avoid the influence of the mean drift from the computations and take into consideration directly the cloud size. This is reflected into the fact that the average curve depicted in black in Fig 3d tends to present a less steep slope suggesting the presence of a plateau for longer times. On the contrary, the average curve of Fig 3a shows a persistently increasing total absolute dispersion. Fig 3e shows the presence of a periodicity analogue to Fig 3b. However, the total absolute dispersion does not reach values so high since the mean drift is disregarded by the calculations. Fig 3f shows the function *σ*(*t*) for *d*^2^(*t*). The periodicity is thus damped due the absence of mean drift.

### 4.3 Multiple statistics: The interaction of the flow scales and the basin dimensions

Relative dispersion regimes typically depend on the initial separation *r*_0_ and on the forcing injection scale in the flow *r*_*I*_. In particular it is possible to recollect the following:

$$\begin{array}{c} K^{\left(2\right)}\propto r^{2}\;r_{0}<r_{I};\;K^{\left(2\right)}\propto (r^{2}{)}^{2/3}\;r_{0}\geq r_{I};\;K^{\left(2\right)}=const\;r_{0}\gg r_{I} \end{array}$$ (9)

where *r*_*I*_ can be regarded as the Rossby radius of deformation and *r* is the separation. The first regime belongs to the enstrophy cascade in two-dimensional turbulence and corresponds to non-local dispersion where the particles are advected by structures greater than the initial separation. The second regime belongs to the energy cascade where the celebrated Richardson law is recovered. Particles disperse randomly in the third regime since they belong to different flow structures. Therefore, relative dispersion should behave as

$$\begin{array}{c} r^{2}\propto \text{exp}\left(\alpha \chi^{⅓}t\right)\;r_{0}<r_{I};\;r^{2}\propto t^{3}\;r_{0}\geq r_{I} \end{array}$$ (10)

where *α* is of order one and *χ* is the rate at which enstrophy is transferred to scales shorter than *r*_*I*_. [1] evaluated the Rossby radius of deformation from numerical models in the Gulf of Trieste to be 2.16±0.5km during spring. This length is considered as the injection scale in the present work.

**Fig 4:**
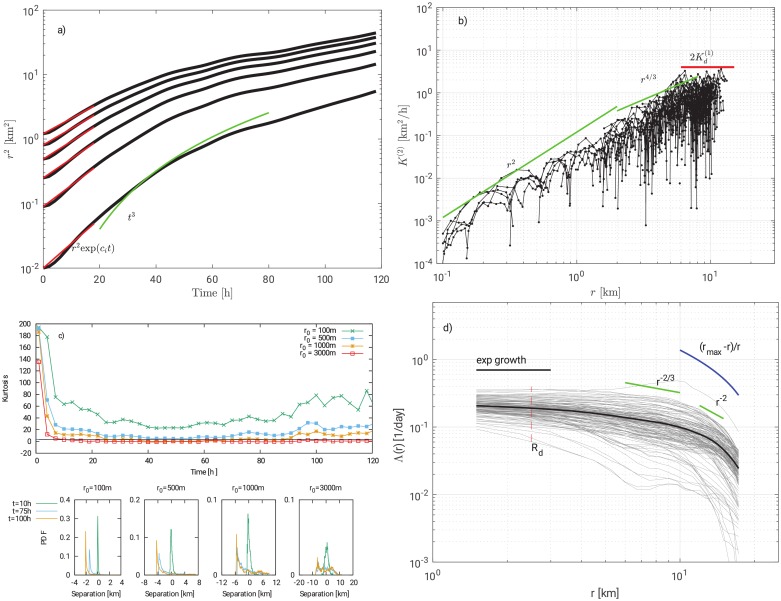
Panel a) shows total relative dispersion *r*^2^. Red curves are best fit of the exponential growth. The green curve is the theoretical cubic growth. Panel b) shows total relative diffusivity *K*^(2)^(*t*). Both linear and constant behaviours are present. Panel c) shows p.d.f. and kurtosis of particles’ displacement. Panel d) shows FSLE values as a function of separation *r*. Thin gray lines are evaluated for different initial conditions (starting time), while the thick black line represents the averaged value over all the simulations. The vertical red line represents the radius of deformation *R*_*d*_.

Fig 4a shows the total relative dispersion *r*^2^ in logarithmic scale in order to visualize a linear relation of the type ln*r*_*i*_ = ln*r*_0_ + *c*_*i*_*t*, where *c*_*i*_ = *αχ*^⅓^. The procedure of changing recursively the initial condition is employed and the black curves represent the average trends. The other curves are omitted in favour of readability. Red curves are the best fit over the first 20h with values of *c*_*i*_ ranging from 0.098 to 0.157 h^−1^.

Relative diffusivity is plotted in Fig 4b with the theoretical trends. Additional information on dispersion can be gathered from p.d.f. of particles’ displacement shown in Fig 4c. At the increase of the initial separation the kurtosis tends to decrease. Separations smaller than *r*_*I*_ show large values of kurtosis, whereas at larger separations the kurtosis tends toward Gaussianity (kurtosis equal to 3). Particles that undergo a random walk lead to Gaussian p.d.f. characterized by such a kurtosis. Particle pairs initially separated by a distance smaller than *r*_*I*_ will experience dispersion that grows exponentially and cubically in time. On the contrary, particles pairs initially separated by a distance greater than *r*_*I*_ will directly experience a cubic growth. It is possible to argue that the first case is a direct consequence of vortex merging [27]. Fig 1 shows a sequence of vortex merging captured through the swirling strength. The velocity gradient tensor in two dimensions can have two real eigenvalues or a pair of complex conjugate eigenvalues [28]. Complex eigenvalues are associated with a spiral motions. Therefore, vortices are easily identified by plotting iso-regions of the complex eigenvalue.

FSLE Λ(*r*) curves are here evaluated through original pairs. Since the measured velocity fields have a 1.5 km resolution in space, smaller scales will not be considered. Following [29] we evaluated the curves for several different initial conditions, as in the case of absolute dispersion. The trends show the existence of an exponential regime with values ranging between 0.12 and 0.37 day^−1^. The average strength is approximately 0.22 day^−1^, whose order of magnitude is in agreement with the findings of [29] and [30] on analogous length scales. The average curve in Fig 4d shows that the exponential growth is limited to scales not greater than 2 km, in agreement with the estimated deformation radius. Furthermore, as the separation *r* increases, the FSLE slope suggests the presence of both the Richardson regime (∼ 6 < *r* <∼ 10 km) and the linear regime (∼ 10 < *r* <∼ 13 km).

For larger spatial scales, as pointed out by [31], the behavior of FSLE curves should follow the theoretical one $\Lambda \left(r\right)\propto \frac{r_{max}-r}{r}$ when approaching length scales of the maximum dimension of the basin *r*_*max*_. Such a behavior is shown in Fig 4d with a blue curve where the spurious subdiffusive regime is a result of the boundary constraints.

## 5 Conclusions

Evaluation of absolute and relative dispersion is of great importance in order to assess the mixing properties of a basin. However, the evaluation of absolute dispersion is not independent from the initial conditions. This means that a specific evaluation of the time scale over which initial conditions are lost must be considered. This work shows a significant influence of the initial conditions on the absolute dispersion. It is possible to argue that a strong forcing that influences initial conditions is the tide. This can be deduced by the analysis of the velocity auto-correlations that tend to decrease for long times, as expected, but show a persistent and periodic, even if small, correlation. Usually, initial conditions are considered to be lost after a time equal to the Lagrangian Integral Time Scale *T*_*L*_, i.e. the integral of velocity auto-correlations. Besides, *T*_*L*_ defines when the behaviour of absolute dispersion changes from a quadratic function of time to a linear function of time. However, it is possible to observe from this work the persistent importance of initial conditions over absolute dispersion even for long times. As a result, it is possible to recover another time scale associated to the fading of the influence of the initial conditions over the absolute dispersion. Such a time scale is evaluated with the function *σ*(*t*). This should be interpreted as a quantity that measures how strong could be the effect of initial conditions with respect to an average value. *σ*(*t*) could well be adopted in order to compare the absolute dispersion computed in different ways. The smaller *σ*(*t*) is as time elapses, the weaker initial conditions are on the outcome of absolute dispersion computations. This measure can well be evaluated alongside with *T*_*L*_ in order to further characterize the mixing properties of a basin.

Finally, relative dispersion is evaluated both as a function of the separation and as Finite-Size Lyapunov Exponent (FSLE) curves in order to unravel the several mixing regimes of the Gulf of Trieste. The approach pursued is the same: relative dispersion and FSLE are evaluated at the varying of the initial conditions and multiple results are obtained. Averages are evaluated and a mean behaviour is shown.
