# Correction: Influence of initial conditions on absolute and relative dispersion in semi-enclosed basins

**DOI:** 10.1371/journal.pone.0221009

**Published:** 2019-08-06

**Authors:** The PLOS ONE Staff

The Data Availability statement for this paper is incorrect. The publisher apologizes for this error.

The correct Data Availability statement is: This research work relies on surface velocity fields that can be freely downloaded from the following repository: Magaldi, Marcello G., Mantovani, Carlo, and Cosoli, Simone (2019). Current velocities measured by HF radars in Gulf of Trieste—April 2012 [Data set]. Zenodo. http://doi.org/10.5281/zenodo.3245408
